# Unraveling the Molecular Inhibition and Conformational Changes of Hsp70 and Hsc70 Induced by VER-155008, a Competitive ATPase Inhibitor through Molecular Dynamics Simulations and Principal Component Analysis

**DOI:** 10.1021/acsomega.5c05832

**Published:** 2025-12-17

**Authors:** Maria Caroline Barbosa da Silva, Carlos Gefferson Silva Falabelo, Elvis Santos Leonardo, Claudir Oliveira, Renan Patrick da Penha Valente, Anderson H. Lima, Sérgio A. de Souza Farias, Khayth Nagata, Kauê Santana da Costa, Paulo Sérgio Taube

**Affiliations:** † Laboratório de Simulação Computacional, Instituto de Biodiversidade, 245076 Universidade Federal do Oeste do Pará, Rua Vera Paz, Salé s/n, 68040-255 Santarém, Pará, Brazil; ‡ Laboratório de Simulação Computacional, Instituto de Ciências da Educação, Universidade Federal do Oeste do Pará, 68040-255 Santarém, Pará, Brazil; § Laboratory of Computational Simulation and Molecular Modeling, Instituto Nacional de Pesquisas da Amazônia (INPA), 69067-375 Manaus, Amazonas, Brazil; ∥ Laboratório de Planejamento e Desenvolvimento de Fármacos, Instituto de Ciências Exatas e Naturais, 37871 Universidade Federal do Pará, Rua Augusto Corrêa, 01, 119, 66075-110 Belém, Brazil

## Abstract

The heat shock protein 70 kDa (Hsp70) is critical for the survival of cancer cells, playing a role in developing chemotherapy resistance, since it inhibits apoptosis of these cells and ensures their survival in stressful environments. Due to its structural similarity with heat shock cognate 70 kDa (Hsc70), the design of new selective Hsp70 inhibitors presents significant challenges. Previous studies have reported that the molecule VER-155008 functions as a nonselective inhibitor of Hsp70 by binding to the nucleotide-binding domain of both proteins, thereby acting as a competitive inhibitor of adenosine triphosphate (ATP) binding. In the present study, molecular dynamics (MD) simulations and free energy landscape (FEL) analysis were used to investigate the conformational dynamics of Hsp70 and Hsc70 with the competitive ATPase inhibitor VER-155008, revealing its binding mechanism and its role in inducing a half-open conformation that inhibits ATP binding. Our findings highlight key residues Ser275, Lys271, and Glu268 involved in the stabilization of the inhibitor binding and some conformational states in both proteins due to the inhibitor binding, explaining molecular characteristics that could be used to develop new selective inhibitors at the nucleotide binding site of Hsp70, thus aiming to advance the development of targeted therapies in cancer treatment.

## Graphical abstract

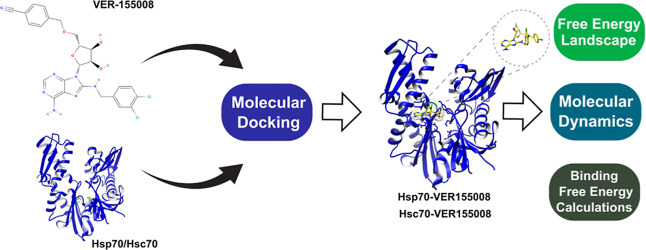

## Introduction

The heat shock protein 70 kDa (Hsp70) plays a crucial role in cancer cells’ survival and is relevant to the development of resistance to chemotherapies. Its ability to protect against cellular stress and prevent apoptosis makes it an important therapeutic target.^1,2^ However, developing selective inhibitors for Hsp70 is challenging due to its high structural similarity to the heat shock cognate 70 kDa protein (Hsc70), a constitutive protein form essential for normal cellular functions. Hsc70 has 85% sequence identity with Hsp70 and represents a constitutively expressed cognate protein from the Hsp70 family.^3^ It is the main maintenance protein of this family and can represent up to 1% of the total cellular protein content, with possibly higher levels in transformed cells. It is involved in many functions similar to those of Hsp70, making it an essential protein for cancer cell survival.^4–9^

The Hsp70 protein family contains two functional domains: the first is named the nucleotide-binding domain (NBD) and it is located at the N-terminal region, and the second one is the substrate-binding domain (SBD, C-terminal region). The nucleotide-binding domain performs ATPase activity and is subdivided into four subdomains, partitioned into two lobes named I and II. In contrast, the substrate-binding domain is subdivided into α and β subdomains.^5,10–12^

In Hsp70, ATP binds to the NBD located in the cleft formed between NBD lobes I and II. This site lies between the IA/IB and IIA/IIB subdomains and contains the nucleotide-binding motifs Walker A/P-loop and Walker B. These motifs are integral parts of the site that coordinates the phosphates and Mg^2+^ and are essential for nucleotide binding. In addition, the SBD shifts, and parts of it—particularly the β-sheet and the α-helical lid—are rearranged relative to the NBD when ATP occupies this pocket, promoting an “open” conformation with low substrate affinity.^13,14^

Acidic amino acids such as Asp10, Glu175, Asp199, and Asp206 have been shown to influence ATPase kinetics, indicating a functional role for these residues within or near the site. Glu171 has been identified as critical for the allosteric coupling between ATPase activity and substrate binding in the SBD, meaning it is essential for properly translating ATP hydrolysis into changes in SBD affinity.^15^

Although different druggable sites have been characterized in the NBD, using the Hsp70 as a molecular drug target has proven extremely challenging because ATP/ADP almost constantly occupies the NBD during the exchange process.^16–18^ This prevents drug-like compounds from effectively binding to the pocket and acting as Hsp70 inhibitors through direct competition with the NBD.^18^ Furthermore, Hsp70 shares close structural similarities with other members of the Hsp70 family, particularly Hsc70, a constitutively expressed protein whose activity is essential for normal cellular function.^19^

The human Hsp70 family comprises multiple isoforms, such as HSPA1A, HSPA1L/Hsp70-hom, HSPA2/Hsp70-2, HSPA5/BiP/GRP78, HSPA6/Hsp70B′, and HSPA8/Hsc70, each with distinct structural specificities. The NBDs of HSPA1L, HSPA2, HSPA5, and HSPA6 share approximately 67–92% structural similarity with the NBD of HSPA1A, the major stress-inducible isoform. However, notable structural differences exist among them, particularly in HSPA5, which stands out as the most structurally divergent isoform. This protein is the least conserved member of the family in terms of its NBD structure, showing that when bound to ADP, the nucleotide-binding cleft becomes slightly open.^17^

The presence of inorganic phosphate in the active sites of HSPA1A, HSPA1L, HSPA2, and HSPA6 suggests that these isoforms undergo ATP hydrolysis, whereas HSPA5 retains ADP and a metal ion but no inorganic phosphate.^17^ HSPA8 shares approximately 85% sequence identity with HSPA1A; both exhibit the NBD–linker–SBDβ–SBDα architecture, differing mainly in their dynamics and sensitivity to covalent modifications.^20^ The nucleotide-binding domains of human Hsp70s are primarily responsible for the conserved ATPase mechanisms. These structural distinctions explain the selectivity of inhibitors toward Hsp70 and its isoforms.^17,20^

Previous studies reported that a molecule named VER-155008 (IUPAC name: 4-[[(2*R*,3*S*,4*R*,5*R*)-5-[6-amino-8-[(3,4-dichlorophenyl)­methylamino]­purin-9-yl]-3,4-dihydroxy-oxolan-2-yl]­methoxymethyl]­benzonitrile), act as inhibitor of Hsp70 expressed in cancer cells.^21,22^ Schlecht et al. (2013) showed that VER-155008 binds to the NBD of Hsp70 leading to the semiopen conformation of the Hsp70, thus acting as a competitive inhibitor of the ATP binding. However, studies have demonstrated that it is not specific to only one Hsp70 isoform.^11^

In the NBD of Hsp70, the residues Lys271 and Ser275 play a crucial role in establishing hydrogen bond interactions with VER-155008.^16^ Furthermore, Arg272 is engaged in π-stacking interactions with the dichlorobenzene moiety of the inhibitor, which aids in the proper orientation and binding of the compound to the NBD of Hsp70.^16^ The molecular function of Hsp70 is closely linked to its ATPase activity, which drives conformational changes necessary for its chaperone functions.^16,23,24^ It has been demonstrated that ATPase competitive inhibitors of Hsp70 induce conformational changes that might effectively disrupt its ability to hydrolyze ATP, thereby impairing its function.^25^ The analyses of conformational changes in the proteins induced by ATP/ADP-competitive inhibitors consist of an interesting strategy to block the Hsp70 protein isoforms.^26^ In the present study, we performed molecular dynamics (MD) simulations to explore the molecular details of interactions and the selectivity of VER-155008 against Hsp70 and Hsc70. We analyzed the conformational changes of both proteins induced by inhibitor binding over time and combined our analysis with the free energy landscape (FEL) approach. The study aims to elucidate the molecular mechanisms underlying the inhibition of Hsp70 and Hsc70 by VER-155008.

## Computational Methods

An overview of the applied computational workflow in the study is represented in Figure 1.

**Figure 1:**
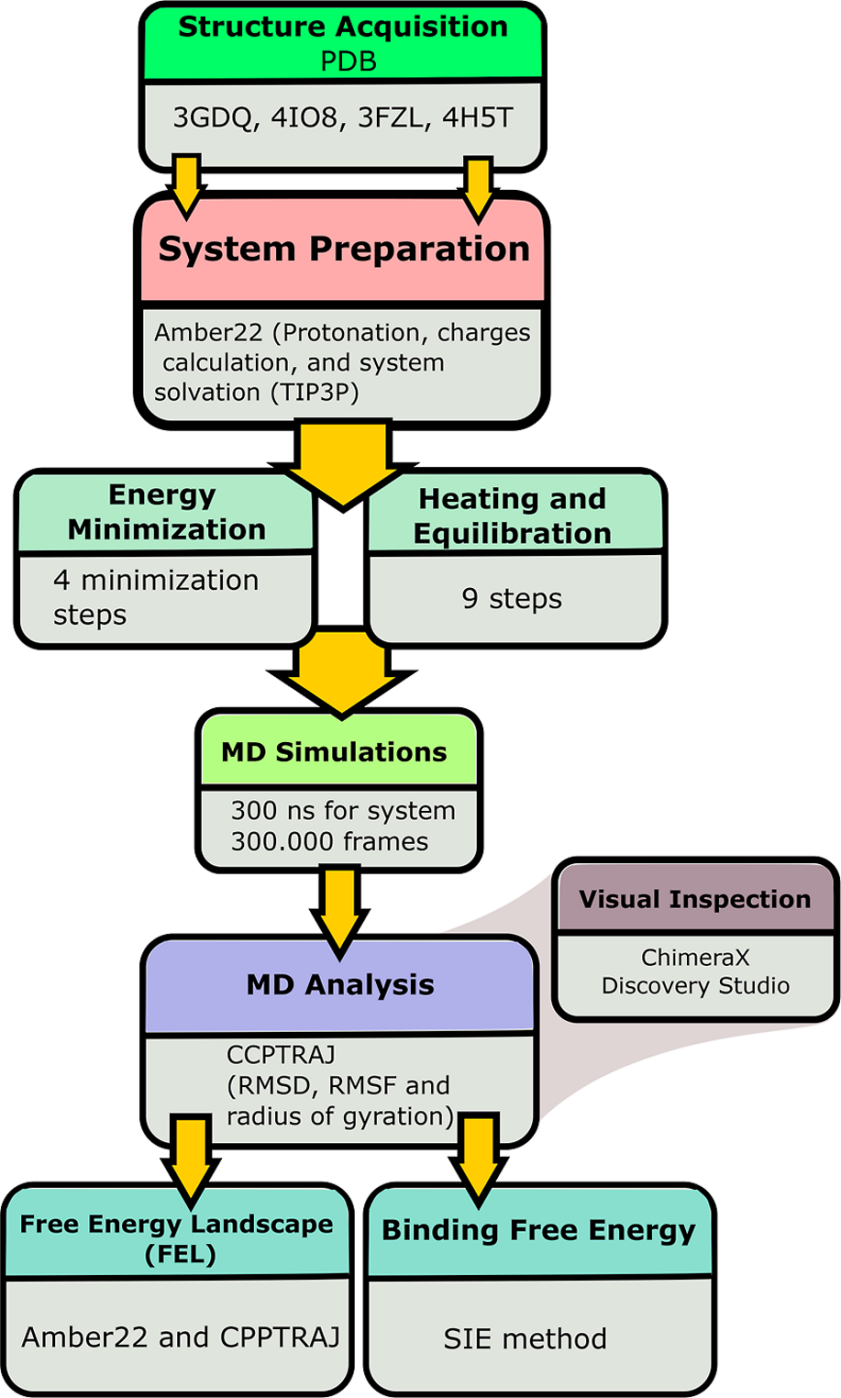
Schematic representation of the computational procedure applied in the present study.

### Molecular Dynamics Simulations

To analyze the induced conformational changes over time in the Hsp70 and Hsc70 and to examine their intermolecular interactions when complexed with the VER-155008, molecular dynamics (MD) simulations were performed with a total time of 300 ns using the Amber22 package.^27^ obtaining 300,000 frames from the simulation. A total of three Hsp70 systems and three Hsc70 systems were analyzed: Hsp70 in its apo form (ligand-free), Hsp70 in holo form (complexed with adenosine diphosphate, ADP), Hsp70 complexed with VER-155008, Hsc70 in its apo form, Hsc70 complexed with ADP, and Hsc70 complexed with VER-155008.

The coordinates of the VER-155008 complexed with Hsp70 were obtained from the structure available under the PDB code: 4IO8 (X-ray diffraction, resolution: 2.58 Å), and the coordinates of VER-155008 complexed with Hsc70 were obtained from the PDB code: 3FZL (X-ray diffraction, resolution: 2.20 Å). The coordinates of the ADP complexed with Hsp70 were obtained from the PDB code 3GDQ (resolution: 1.80 Å, X-ray diffraction). The Hsp70 complexed with ADP was obtained from the PDB code 4H5T (X-ray diffraction, resolution: 1.90 Å). The isoforms modeled for the simulations were HSPA1A for the Hsp70 protein complexed with VER-155008, HSPA8 for the Hsc70 protein complexed with VER-155008, and HSPA1L for the Hsp70 protein complexed with ADP.

First, the protonation state of the ionizable residues was analyzed by p*K*_a_ calculations using the PDB 2PQR server^28^ using the Poisson–Boltzmann model.^29^ Then, the ligand charges were calculated using the restrained electrostatic potentials with the Hartree–Fock method^30^ and the 6-31G* basis set in the Gaussian 09 software.^31^ The tLeap module of Amber22 was used to build the receptor–ligand complex parameters, where the ff19SB force field^32^ described the protein atoms, and the general Amber force field treated the ligand atoms.^32^ Finally, these complexes were solvated in a truncated octahedron water box using an explicit solvation model, TIP3P.^33^ A radius of 12.0 Å was set between the water box wall and the protein surface. To neutralize the Hsp70 and Hsc70 systems, we added counterions: 1 Na^+^ to the Hsp70 and 2 Na^+^ to the Hsc70 complexes.

Each analyzed Hsp70 and Hsc70 system was minimized to reduce the overall energy using the conjugate gradient and steepest-descent algorithms.^34^ Minimization was performed in four steps: the first included the solvation waters and counterions; the second corresponded to the protein’s hydrogen atoms; the third corresponded to the hydrogens and water molecules; and finally, the fourth step included the entire solvated protein–ligand complex system.

Then, the Hsp70 and Hsc70 systems were heated from 10 to 300 K over 100 ps at constant volume. A total of 200 ps of density equilibration with weak restraints was performed for the Hsp/Hsc70–ligand complexes, followed by 700 ps of constant pressure equilibration at 300 K. The Langevin thermostat was used to maintain the temperature at 300 K. The SHAKE^35^ was used to constrain all hydrogens, and we used an integration time step of 2 fs. Then, 300 ns of MD simulations were performed for each Hsp/Hsc70 system under an isothermal–isobaric (*NPT*) ensemble. The molecular dynamics trajectories were analyzed using the root-mean-square deviation (RMSD) and radius of gyration (*R*_g_) over the simulation time, and the fluctuations of the amino acid residues were assessed through the root-mean-square fluctuation (RMSF) values obtained from the C_α_, N, and O atoms of the polypeptide backbone. To analyze the conformational stability of the complex over time, RMSD analysis was also performed on the protein.

To calculate the RMSD, the coordinates of the structures over time were aligned with the initial structure to minimize the impact of global movements. These values were then plotted as a function of time. This analysis enables the comparison of regions exhibiting high conformational fluctuations throughout the simulation.^36^ To calculate the RMSF, the fluctuations of the heavy atoms of the polypeptide chain relative to the average positions were considered over time. The ΔRMSF was plotted against residue positions of the protein sequence, enabling the identification of regions with greater flexibility and thus providing a better understanding of the protein’s dynamics.^36^ The ΔRMSF values were obtained by subtracting the RMSF of the complexes from the unbound (ligand-free) state. The radius of gyration (*R*_g_) analysis was performed to assess the compactness of the protein structures over time. The radius of gyration is a measure that quantifies the distribution of atoms relative to the center of mass.^37^ The *R*_g_ plots were generated by plotting the values over time, allowing for the observation of conformational changes related to the compactness of the analyzed proteins during the simulations.^36^ The RMSD, RMSF, and *R*_g_ values were extracted for the last 30 ns of the MD trajectory using the Cpptraj of Amber22.^38,39^ The values were plotted using the Python programming language and the Matplotlib library.^40^

### Binding Free Energy Calculations

The trajectories of each molecular dynamics (MD) simulation were used as a starting point to calculate the binding energy of the human Hsp70 complex with the inhibitors VER-155008. The energies were calculated using the solvated interaction energy (SIE) method available in the Sietraj package^41,42^ was used to calculate the binding free energy of the protein–ligand complexes. Cpptraj was used to extract the last 30 frames of the stable RMSD values (plateau regions) for energy calculations. Then, ions and water molecules were omitted to perform the binding energy calculations.

The SIE is calculated for each frame using rigid separation between the target and the ligand. The SIE is the sum of the van der Waals and Coulomb intermolecular interactions, along with the change in reaction field energy and nonpolar solvation energy. The SIE value is then scaled by an empirically determined factor, obtained from a data set of 99 protein–ligand complexes.

Additionally, the experimental binding free energy values were calculated from the inhibition constant values (IC_50_) obtained for each inhibitor using eq 1, using the gas constant and the temperature^43^

1 $$\Delta G=-RT\;\ln K_{i}$$

where Δ*G* corresponds to Gibbs binding free energy, and *K*_i_ to the inhibition constant. The Gibbs free energy is a thermodynamic state function that combines enthalpy and entropy, and it represents the maximum amount of useful work that a system can perform under constant temperature and pressure conditions.^44^ The *K*_i_ of the VER-155008 was obtained from the previous study.^16^

### Free Energy Landscape

The free energy landscape (FEL) projection was performed using the Cpptraj from the AmberTools package.^45^ Initially, principal component (PC) analysis was applied to identify the main modes of protein motion throughout the molecular dynamics (MD) simulation. This approach reduces data dimensionality and highlights collective fluctuation patterns of amino acids based on the α carbon (C_α_)^46^ atoms. Principal component (PC) analysis is a statistical technique used to reduce the dimensionality of complex data sets while preserving the most important variability in the data. Herein, PC was applied to the atomic coordinate trajectories to identify the dominant modes of motion that contribute most to conformational changes, providing valuable insights into the protein’s dynamic behavior associated with the energy landscape.^47^ These techniques have been widely applied to identify major conformational shifts relevant to function, stability, or binding interactions, as well as structural flexibility in proteins.^48,49^

First, the average fluctuation of C_α_ atoms in the Hsp70 and Hsc70 systems was calculated as a reference for the covariance matrix projection.^50^ After preprocessing, the covariance matrix of the *XYZ* Cartesian coordinates was constructed, centered on the mean of the C_α_ atoms. The diagonalization of this matrix provided the eigenvectors and their corresponding eigenvalues, representing the dominant motion patterns of the protein. The two principal components (PC1 and PC2) were analyzed, as they capture most of the structural variation in the *XYZ* coordinates.^50^

The FEL for each state was calculated based on the second law of thermodynamics, which establishes that systems naturally tend to evolve toward states of higher probability and lower free energy,^51^ associating state probability with the principal components (PC1 × PC2).^52,53^ Free energy (FE) was calculated using the Boltzmann equation: *F* = −*k*_B_ *T* ln­(*P*). The centroid of conformational states was determined by clustering in UCSF Chimera^54^ facilitating the analysis of predominant conformational motions. This approach enabled the identification of protein regions with greater flexibility and potential structural changes related to their biological function.^55^

## Results and Discussion

Hsp70 is a chaperone protein vital for the survival of tumor cells, which has led to the development of inhibitors targeting its mode of action.^56^ These inhibitors work by either preventing the interaction of Hsp with their cochaperones^57^ or by covering the crucial ATPase binding pocket needed for Hsp70s activation.^16^ One such compound, VER-155008, has been identified as a potential ATP competitive inhibitor that binds to Hsp70, inhibiting ATP hydrolysis. It has also demonstrated anticancer properties across multiple cancer types.^23^ Molecular dynamics have been widely applied to elucidate the conformational mechanism of protein catalysis^58^ and the conformational changes induced in proteins by the inhibitor and substrate binding.^48,59,60^ Herein, we performed MD simulations to explore the conformational dynamics of inhibition of Hsp70 and Hsc70 related to the VER-155008 binding. Then, we performed binding free energy calculations and energy decomposition per residue to investigate details of intermolecular interactions related to the inhibition and induced conformational changes in the proteins.

We noticed that after 80 ns of simulation, the molecular systems of Hsp70 and the unbound state of Hsc70 reached stabilization over time, as demonstrated by the plateau of the RMSD plots of both simulations. In contrast, it was not noticed for Hsc70 in complex with VER-155008. The RMSD values obtained over time for MD of Hsp70 (Figure 2, panel A) exhibit that in the first 20 ns, the RMSD of Hsp70–VER-155008 shows considerable fluctuations, peaking at up to 3.5 Å, suggesting an initial adaptation to the presence of the inhibitor. Then, the RMSD stabilizes at around 2 Å, reflecting a more stable protein conformation in complex with the inhibitor. In contrast, Hsp70 in the unbound state (ligand-free) exhibits a more consistent behavior, with smaller fluctuations and an average RMSD of approximately 1.5 Å, indicating a more rigid structure less affected by dynamic conformations. The RMSD plot of Hsc70–VER-155008 reveals that it initially increases, then quickly reaches peaks up to approximately 4 Å (Figure 2, panel B). After this initial phase, the RMSD stabilizes at around 3 Å. Hsc70 without the inhibitor, on the other hand, shows minimal fluctuation in the RMSD, remaining at around 2 Å. This analysis of the RMSD plots indicates that, although both proteins show a temporal increase in instability when interacting with the inhibitor, Hsc70 shows a more pronounced fluctuation behavior, possibly related to an adaptive conformational response to the presence of VER-155008. These patterns suggest that introducing the inhibitor significantly impacts the stability of Hsc70, more than the Hsp70.

**Figure 2:**
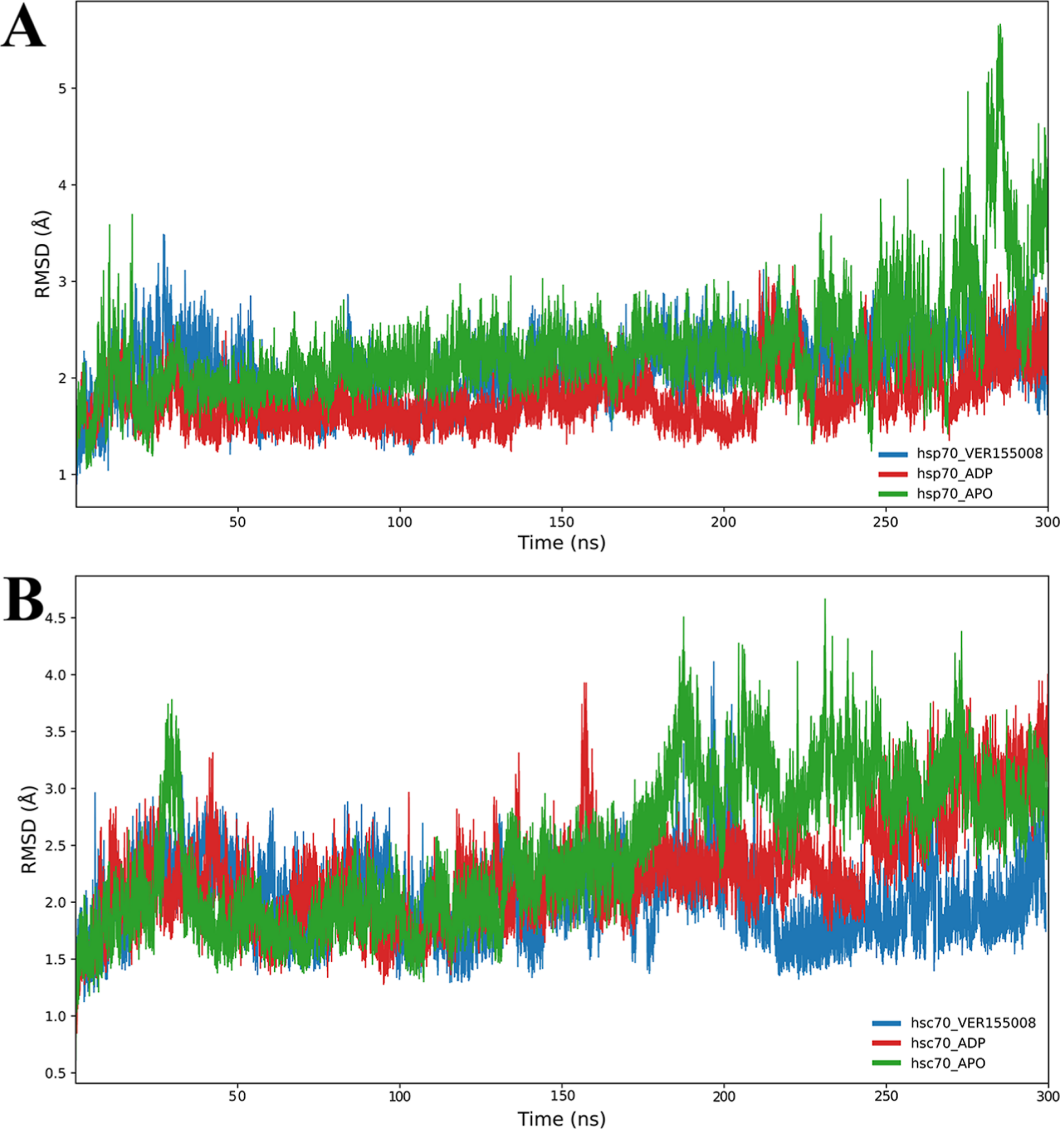
Root mean square deviation (RMSD) plots of the Hsp70 and Hsc70 analyzed over 300 ns of molecular dynamics. Panel A illustrates the variation in the RMSD of Hsp70, where the red line represents Hsp70 complexed with the inhibitor VER-155008, the blue line represents Hsp70 in apo form, and the red line represents Hsp70 in holo form complexed with ADP. Panel B shows the RMSD values of Hsc70, with the red line representing Hsc70 complexed with the inhibitor and the blue line representing Hsc70 in the unbound state (ligand-free). The plots indicate fluctuations and stabilizations in the RMSD values, reflecting the influence of the inhibitor on the conformational dynamics of both proteins.

### Molecular Interactions Formed between the VER-155008 and the ATPase Binding Site of Hsp70 and Hsc70

Previous studies have demonstrated that VER-155008 acts as a selective inhibitor of the Hsp family, including Hsp70 and its homologue protein, Hsc70.^11,61^ The VER-155008 binds to Hsp70s NBD, stabilizing it in a half-open state conformation. This action makes it an ATP/ADP-competitive inhibitor, disrupting the allosteric regulation between the NBD and the SBD.^11,62^ In the present study, the subdomains IA and IIA of NBD of Hsp70 and Hsc70 structures were retrieved from the Protein Data Bank (PDB). Figure 2 shows key residues involved in the intermolecular interactions between VER-155008 and Hsp70 and Hsc70. It is interesting to note that previous studies show no selective binding against Hsc70 and Hsp70, which allows for a comparative analysis of their selectivity, and it explains our choice for VER-155008. Other inhibitors of Hsp70, such as the 2-phenylethynesulfonamide (named PES), interact with the SBD in a nonselective, detergent-like manner, thus demonstrating no specificity for the analyzed isoforms.

The residues Ser275, Lys271, and Glu268 at the NBD formed hydrogen bonds with the VER-155008 (Figure 3, panel A). The Ser275 and Glu268 showed the most stable and frequent interactions, exhibiting the highest occupancy values of the last 10 ns of simulation (Table 1). Similarly, we found hydrogen bond interactions for the residues Ser275 and Lys271 in the Hsc70 ATPase binding pocket. It is interesting to note that the residue Ser275 has been identified as a residue hotspot for Hsp70/Hsc70 binding and an important determinant of selectivity for the initial ATP binding in Hsp70 isoforms.^62^ It was found that the Arg272 (R272) residue is present in the Hsp70 and Hsc70 proteins. However, while in Hsp70 Arg272 forms a π–cation interaction, in Hsc70, there is a hydrogen bond. This difference may indicate that this specific characteristic influences the interaction with the inhibitor. The similar molecular interactions between the inhibitor and Hsc70 residues indicate that, although the proteins share high sequence identity, the differences in their interactions can be exploited to develop more selective inhibitors. Additionally, the molecular interactions formed between VER-155008 and Hsp70 could be leveraged to create more selective therapeutic compounds targeting Hsp70, given that Hsc70 has a constitutive expression and is essential for normal cellular functions.^63,64^

**Figure 3:**
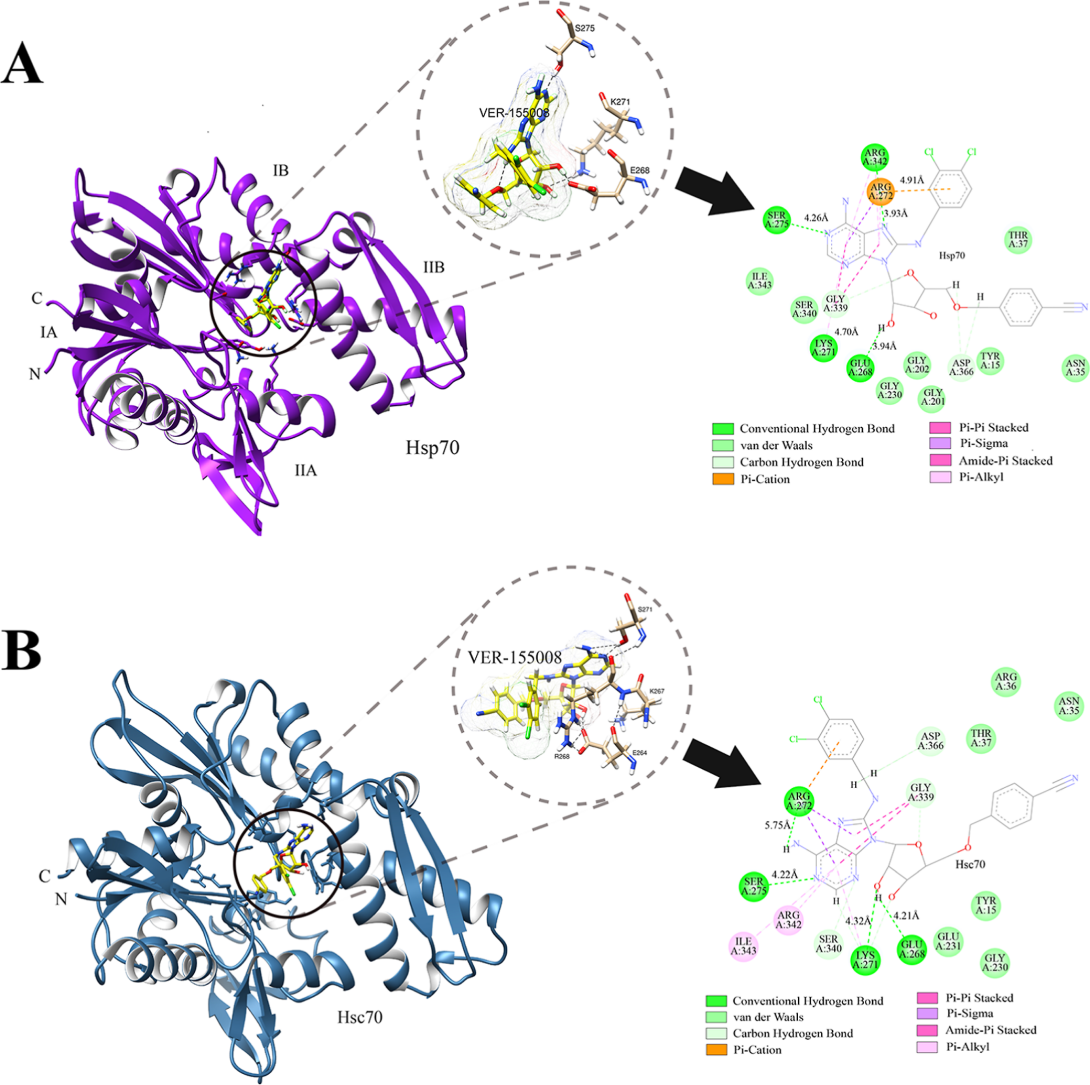
Three-dimensional structures of Hsp70 and Hsc70 complexed with the inhibitor VER-155008 showing the domains and subdomains of both proteins located at N- and C-terminal regions. Panel A shows Hsp70 in purple, highlighting the ATPase binding site complexed with VER-155008 (black circle), interacting with the key residues, such as Ser275 (S275), Lys271 (K271), and Glu268 (E268) involved in the stabilization of the complex. Panel B shows Hsc70 in blue, with the inhibitor VER-155008 highlighted in the black circle, forming interactions with the residues Lys271 (K271), Ser257 (S257), and Glu268 (E268).

**Table 1:** Analyses of Occupancy Obtained in the Last 30 ns of MD Simulations for Both Hsp70 and Hsc70 Complexed with the Inhibitor, VER-155008^a^

donor	acceptor	occupancy (%)	distance (Å)
Hsp70–VER-155008			
Ser275	VER-15008	58.42	4.26
VER-155008	Glu268	100	3.94
Arg342	VER-155008	82.18	3.93
Lys271	VER-155008	18.89	4.70
Hsc70–VER-155008			
VER-155008	Glu268	100	4.21
Ser275	VER-155008	20	4.22
Lys271	VER-155008	64	5.75

a The occupancies represent the interaction frequencies obtained for the complexes over the analyzed simulations.

As demonstrated in previous studies, we found that the adenine moiety of VER-155008 interacts with the Hsp70 nucleotide binding pocket at the region located between the residues Arg272 and Arg342, and forms hydrogen bonds with the oxygen of Ser275 (S275) and the nitrogen (N1) of the adenine ring. The oxygen of the ribose present in VER-155008 (O2) also forms hydrogen bonds with the nitrogen (Nζ) of Lys271 (K271). Additionally, a hydrogen bond is established between the oxygen of the ribose (O3) and a water molecule, as well as with the oxygen (Oδ) of Asp234. The Arg272 (R272) forms a π-stacking interaction with the aromatic ring of VER-155008.

Interestingly, a previous study demonstrated that an inhibitor named sangivamycin 10 forms hydrogen bonds with the adenosine motif, similarly found in the ADP/Pi structure of Hsp70.^26^ Specifically, the pyrrolopyrimidine ring interacts with Ser275, while the ribose’s 2′ and 3′-hydroxyl groups engage with Lys271. Unlike the conformation acquired by ADP/Pi in the NBD of Hsp70, the sangivamycin 10 was found to be crystallized in a relatively more open conformation. The conformation of NBD plays a significant role in the intricacies of the structure–activity relationship observed for nucleotide-derived inhibitors.^65^ The half-open conformation of NBD seen in the ADP/Pi-bound structure is likely influenced by interactions between the β-phosphate group and the two glycine-rich loops in the phosphate-binding region.

### Conformational Changes and Binding Affinities of VER-155008 with Hsp70 and Hsc70

Hsp70s protein family performs their refolding chaperon function by hydrolyzing ATP into adenosine diphosphate (ADP) and inorganic phosphate (Pi), engaging in a complex catalytic cycle that includes several protein conformational shifts.^66^ This process is precisely controlled by various cochaperones, such as heat-shock protein 40 (Hsp40) and the nucleotide exchange factor BAG family molecular chaperone regulator 1. Despite the multifaceted nature of this cycle, presenting several chances to hinder Hsp70s refolding activity, the most straightforward method involves the ATP/ADP-competitive binding of inhibitors to the protein’s conserved nucleotide-binding domain. Thus, analyzing the conformational changes induced by ATP/ADP-competitive inhibitors consists of an interesting strategy to block the protein function.^1^

The higher positive peaks in the ΔRMSF plots indicate that the residues in the bound state of Hsc70 are more prone to larger conformational movements, suggesting greater flexibility compared to the unbound state of the protein (Figure 4, panel A). These findings suggest that Hsp70 adopts a more rigid structure in the absence of the inhibitor. The binding of VER-155008 induces significant conformational fluctuations in Hsc70 residues within the ranges of 50–150 and 300–350. In contrast, analysis of Hsp70 shows that interaction with the inhibitor significantly impacts conformational stability in critical structural regions (Figure 4, panel B). Residues located at the positions between 200 and 300, exhibit highly dynamic behavior in the presence of the inhibitor. This indicates that the inhibitor interaction induces a more pronounced conformational response in Hsc70 compared to Hsp70.

**Figure 4:**
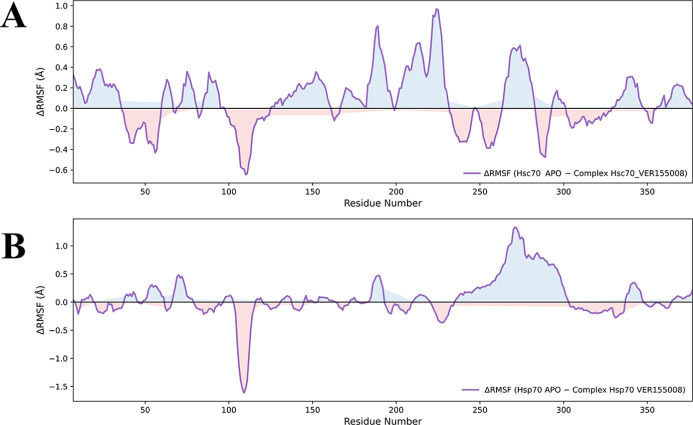
Variation in the root mean square fluctuation (ΔRMSF) values for Hsp70 and Hsc70 complexes compared to the unbound (ligand-free) state. The variation reflects amino acid residues in both proteins with high flexibility over 300 ns of MD simulations. Panel A shows the ΔRMSF values of the Hsc70–VER-155008 complex compared to unbound Hsc70. Panel B shows the ΔRMSF values of the Hsp70–VER-155008 complex compared to unbound Hsp70. For more detailed information on the RMSF raw data, please refer to Supporting Information S1.

The *R*_g_ of Hsp70 in bonded and unbonded states is exhibited in Figure 5, panel A. The blue line represents Hsp70 complexed with VER-155008, while the green line represents Hsp70 in the unbound state. The *R*_g_ values are indicative of the general conformation changes of the protein; lower values suggest a more compact structure, while higher values indicate a more expanded conformation. It can be seen that, during the first 20 ns, Hsp70–VER-155008 exhibits considerable fluctuations, with peaks reaching up to 23 Å, followed by a reduction and stabilization around 22 Å, suggesting that interaction with the inhibitor induces a slight expansion in the protein’s structure. In contrast, Hsp70 in its native form shows a more stable behavior, oscillating around 21.5 to 22 Å, indicating a more compact and stable conformation over time. In Figure 5, panel B, the plots show the *R*_g_ values for Hsc70, with the blue line representing Hsc70 in the presence of the VER-155008 inhibitor and the green line representing Hsc70 without the inhibitor. Hsc70–VER-155008 shows a more dynamic behavior, with peaks that can exceed 23 Å during the simulation, suggesting a more expanded conformation in response to the interaction of the inhibitor. After the initial peak, the turning radius stabilizes, oscillating around 22.5 Å, highlighting a slight expansion compared to Hsc70 without the inhibitor, which remains around 21.75 Å, indicating a relatively compact structure. These spin radius results offer insights into the conformational changes induced by the VER-155008 in the Hsp70 and Hsc70, highlighting relevant differences in the structural dynamics between the two proteins. The Hsc70 appears to be more susceptible to conformational changes in response to the inhibitor, while Hsp70 exhibits greater stability in its conformation.

**Figure 5:**
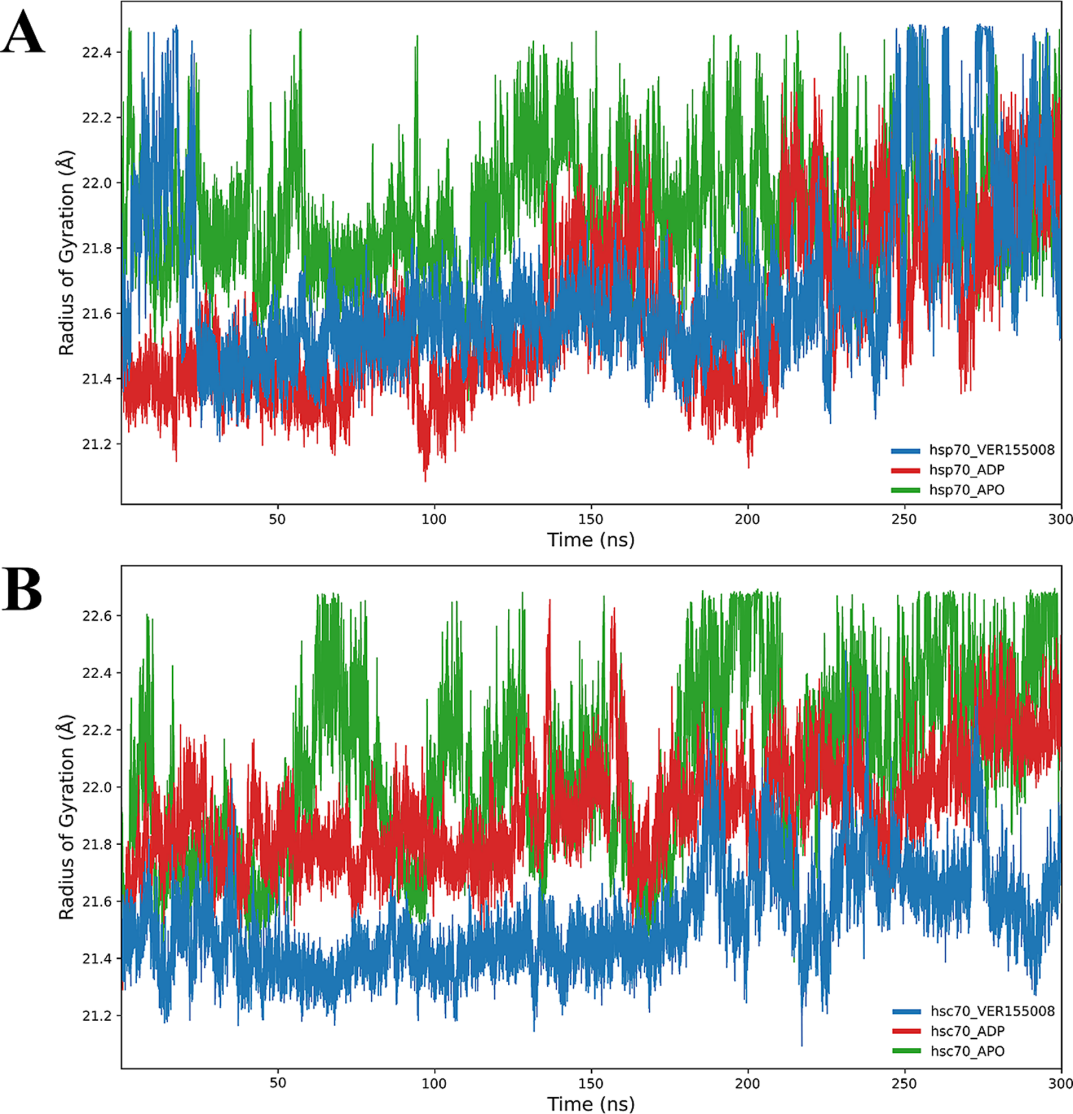
Radius of gyration (*R*_g_) plots of Hsp70 and Hsc70 proteins over 300 ns of MD simulation. Panel A illustrates the *R*_g_ of Hsp70, where the red line represents Hsp70 complexed with the inhibitor VER-155008, and the blue line represents Hsp70 in its native form. Panel B shows the *R*_g_ of Hsc70, with the red line indicating Hsc70 complexed with the inhibitor and the blue line representing Hsc70 without the inhibitor. Fluctuations in the *R*_g_ suggest conformational changes in the protein structures induced by the presence of the inhibitor.

### Binding Affinities and Insights into Competitive Inhibition of Hsp70 by VER-155008

Prior studies show that ATP binding remodels NBD subdomain orientations and lowers SBD substrate affinity; in the present study, however, we restrict our analysis to apo, ADP, and VER-155008 states.^67–69^ The binding of VER-155008 is characterized by the formation of multiple hydrogen bonds with key residues in the ATP/ADP-binding cleft, inducing and stabilizing the half-open conformation of both proteins. This ligand-induced conformational change resulted in a high affinity, showing a binding free energy of −8.47 ± 0.28 kcal·mol^–1^. Similarly, the Hsc70 showed to adopt predominantly a half-open conformation in the presence of the inhibitor, with a binding free energy of −8.93 ± 0.40 kcal·mol^–1^. The results are consistent with the *K*_i_ value of 10.9 ± 2.8 μM obtained for Hsp70 complexed with the inhibitor.^70^ This conformation of Hsp70, observed in the ADP/Pi-bound structure, is likely due to the interaction between the β-phosphate group and two glycine-rich loops in the phosphate-binding region. The ADP showed a binding affinity to the NBS equal to 0.46 ± 0.59 kcal·mol^–1^ for Hsp70. The binding of VER-155008 suggests that it functions as an ATP/ADP-competitive inhibitor, disrupting the allosteric interaction between the two Hsp70 domains, NBD and SBD (Table 2).

**Table 2:** Binding Free Energies Obtained for the Hsp70 and Hsc70 Complexes^a^

binding free energy (Δ*G* in kcal·mol^–1^)	protein–ligand complexes
–8.47	Hsp70–VER-155008
–8.93	Hsc70–VER-155008
0.46	Hsp70–ADP
–5.64	Hsc70–ADP

a The binding energies were calculated using the SIE methods for 30 frames of the MD trajectories.

Studies have shown that ATP binding to the NBD of Hsp70 plays a crucial role in modulating the affinity and kinetics of substrate interactions with the SBD.^71,72^ This process triggers conformational transitions, shifting from a state with high substrate affinity to a lower affinity to the substrate. These structural changes significantly alter the conformational flexibility of the SBD, which is crucial for effective substrate binding and release.^72,73^ For example, a previous study has shown that the Hsp70 of *Escherichia coli* (*Ec*Hsp70) retains a conserved and functionally essential interdomain linker represented by the motif ^389^VLLL^392^ located at the NBD. Key residues form allosteric networks, allowing the NBD to function as a nucleotide-sensitive switch.^73^ The ATP binding induces changes in subdomain orientations and causes long-range perturbations along the subdomain IA and IIA interfaces. In the presence of ATP, the linker associates with the edge of the IIA β-sheet, creating a structural connection between the linker and the NBD.^73^ This establishes an allosteric communication pathway that extends from the ATP-binding site in the NBD to the adjacent SBD through the interdomain motif.^74^

### Free Energy Landscape Reveals Increased Conformational Instability of Hsc70 upon Inhibitor Binding

The free energy landscape (FEL) analysis is a powerful computational technique widely applied to visualize and understand the conformational transitions in proteins.^36,75,76^ The FEL maps the free energy states relative to conformational coordinates, revealing energy minima and maxima that correspond to stable and unstable configurations, respectively. This approach is essential for understanding how proteins fold, interact with other molecules, and perform their biological functions. The identification of low-energy regions in the free energy landscapes (Figure 6) derived from principal component analysis reveals distinct conformational behavior for Hsc70 in complex with VER-155008 (panel A) versus ADP (panel B). Hsc70–VER-155008 samples a broader and more continuous ensemble of low-energy conformations, indicative of increased conformational heterogeneity (higher conformational entropy), whereas Hsc70–ADP occupies fewer, well-defined minima consistent with stabilization of discrete states. Consequently, rather than implying a simple increase in energetic instability, the FELs suggest that VER-155008 promotes structural plasticity, while ADP favors conformational confinement.

**Figure 6:**
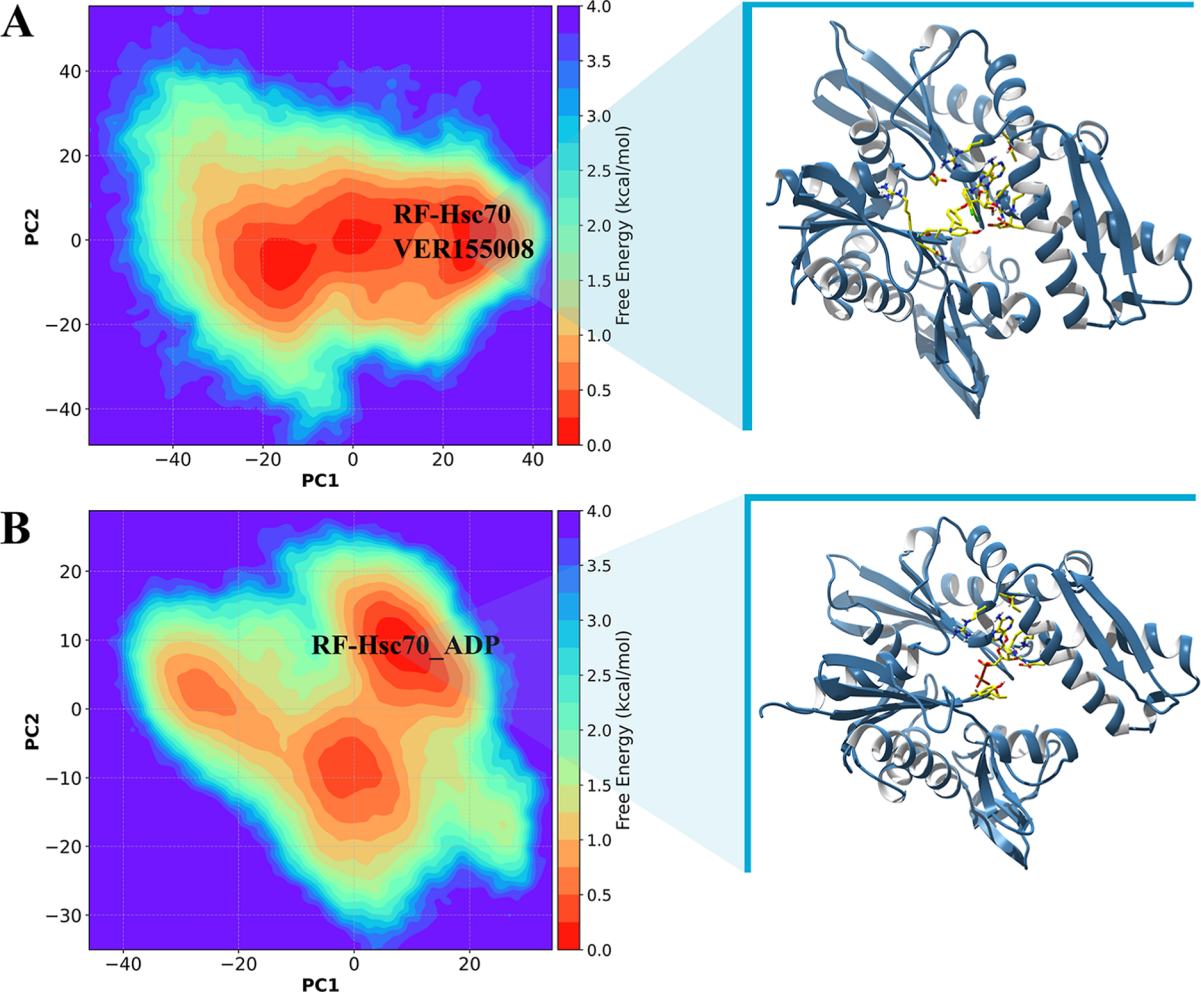
Free energy landscape (FEL) plots for Hsc70–VER-155008 (A) and Hsc70–ADP (B), mapped against the principal components (PC1 and PC2) derived from principal component (PC) analysis.

The free energy landscapes of Hsp70 projected onto PC1 and PC2 (Figure 7) reveal ligand-dependent conformational behavior. In complex with VER-155008 (panel A), Hsp70 samples a broad and relatively continuous ensemble of low-energy conformations, indicative of increased conformational heterogeneity and plasticity. In contrast, the Hsp70–ADP complex (panel B) occupies a smaller number of well-defined minima separated by discernible barriers, consistent with ADP-mediated stabilization of discrete conformational states. These results suggest that VER-155008 promotes structural flexibility of Hsp70, whereas ADP biases the protein toward confined conformational states; quantitative comparison of basin populations and transition kinetics (e.g., via MSM or population Δ*G* estimates) would further clarify the thermodynamic and kinetic implications.

**Figure 7:**
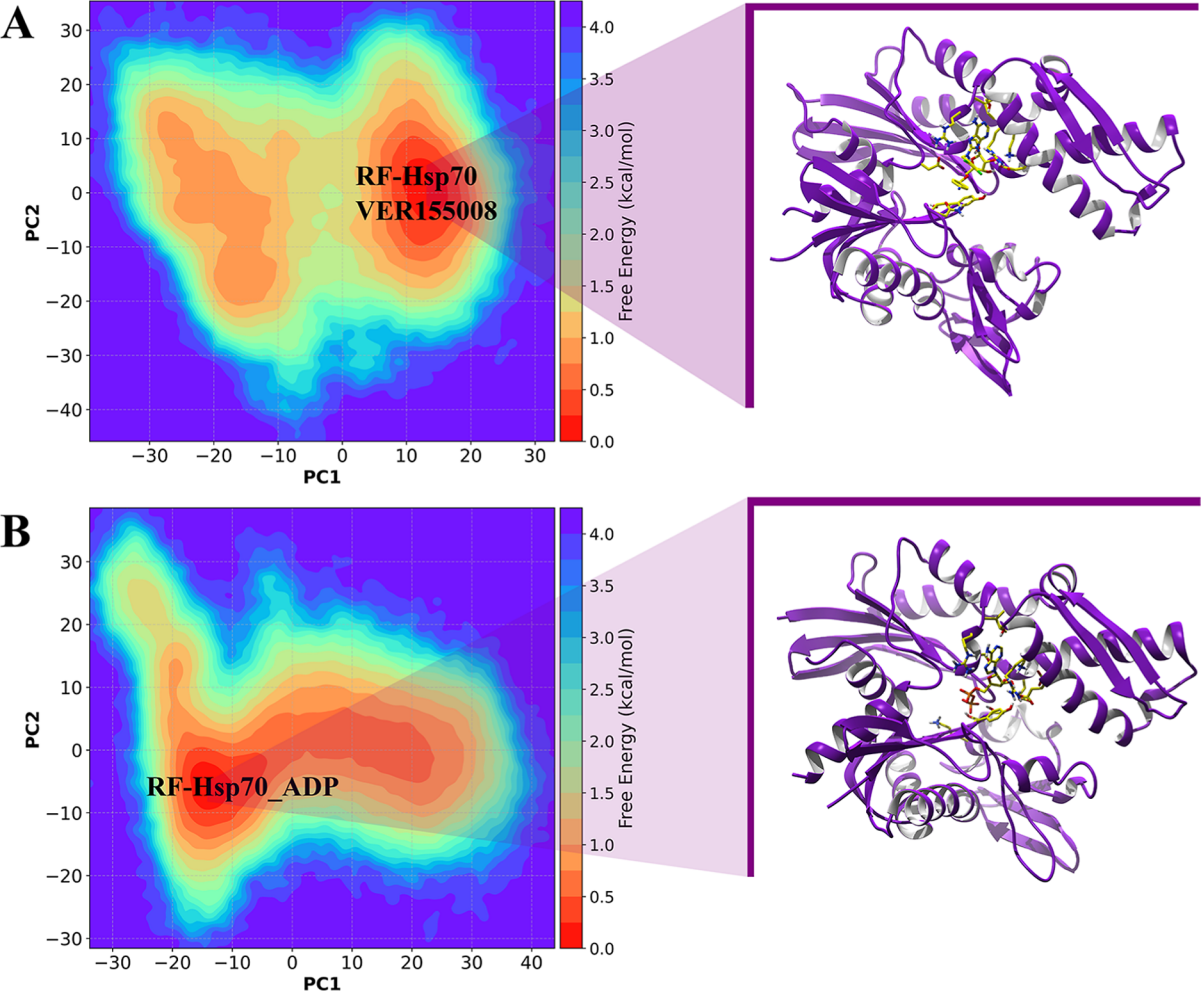
Free energy landscape (FEL) plots for Hsp70–VER-155008 (A) and Hsp70–ADP (B), mapped against the principal components (PC1 and PC2) derived from principal component (PC) analysis.

The FEL surface of Hsc70 exhibits a complex free energy distribution with respect to the two principal components (Figure 6, panel A). The RF-Hsc70 highlight reveals a more prominent energy minimum, with values between ∼1 and 1.5 kcal·mol^–1^, suggesting more stable conformational states. These low-energy conformations are fundamental to protein functionality, indicating the presence of three conformational states for Hsc70. However, transitioning between different conformational minima requires overcoming energy barriers exceeding 3 kcal·mol^–1^. This behavior corroborates previous data, which indicated significant RMSD fluctuations for Hsc70 in the presence of VER-155008, suggesting that the inhibitor induces an increase in free energy and greater conformational instability compared to Hsp70, which is consistent with the conformational changes similarly induced in the protein upon ADP binding.^16^ In panel B, the FEL plot of Hsp70 exhibits a similar topology but distinct characteristics compared to the FEL of Hsc70. The three-dimensional structure of Hsc70 represents a single specific state, in addition to fluctuations between microstates (energy dispersions beyond the minimum). The RF-Hsp70 highlights free energy regions between 1 and 2 kcal·mol^–1^, indicating probable conformational states. However, the energy distribution suggests a lower dispersion of conformational states than Hsc70, but greater variability among microstates. Thus, the three-dimensional structure associated with Hsp70 reflects a set of conformations in dynamic equilibrium, which may indicate greater structural flexibility and lower relative stability. The Hsc70 has larger regions with high conformational variation with energy minima (Figure 6, panel A). In contrast to Hsp70, Hsc70 bound to VER-155008 has a greater closure of the NBD cleft in subdomain IIB toward IB. The conformational changes of subdomain IIB on Hsc70 bound to VER-155008 favor more stable interactions.

It is important to highlight that our decision to perform the MD simulation at 300 K, rather than the physiological temperature of 310 K, was made to balance computational efficiency with the accurate capture of the system’s relevant dynamics. The 300 K is widely used in the literature to evaluate the Hsp70 form of human and other species, and it has been shown to provide reliable insights into protein conformational behavior without significantly compromising biological relevance, inducing conformational instability, or impairing the reproducibility of findings of the in vitro experimentation.^77,78^

The FEL results for Hsc70 and Hsp70 align with previous molecular dynamics data, demonstrating that Hsc70, when bound to the inhibitor, exhibits a high conformational instability, as evidenced by the RMSD and RMSF values obtained over the MD. The rise in free energy highlights the influence of inhibitor interactions on modulating protein conformational transitions.

## Final Considerations

Herein, we performed MD simulation analyses combined with FEL to explore the conformational changes induced in the Hsp70 and Hsc70 over time. These simulations aimed to elucidate the interactions and selectivity of the inhibitor VER-155008 with the Hsp70 protein compared to Hsc70. Hsp70 is essential for cancer cell survival and is involved in the cellular stress response, while Hsc70, sharing 85% sequence identity with Hsp70, is constitutively expressed and involved in maintaining normal cellular functions. The high structural similarity between these two proteins makes developing selective inhibitors challenging. Our study demonstrates that VER-155008 interacts with the residues Ser275, Lys271, and Glu268 located at the nucleotide binding pocket of both proteins, and it has a pronounced impact on the conformational stability of Hsc70, exceeding its effect on Hsp70, an important molecular target in cancer. This indicates that the structure of VER-155008 poses significant challenges for designing more selective inhibitors against Hsp70 when used as a reference compound. Interestingly, using structural comparison, we found that the Arg272 (R272) residue is present in the Hsp70 and Hsc70 proteins. However, while in Hsp70 Arg272 forms a π–cation interaction, suggesting that this specific feature may influence the interaction with the VER-155008 and could be used to design selective inhibitors. Our conclusions are derived exclusively from in silico analyses that included MD simulations, FEL, and binding free-energy calculation thus, they should be interpreted as mechanistic hypotheses that require experimental corroboration. The present study should be viewed as a computational framework that predicts the conformational dynamics of both Hsp/Hsc proteins rather than a substitute for biochemical or cellular validation. It is important to note that the inhibitor VER-155008 has been characterized extensively for human Hsp70/Hsc70 isoforms, but its efficacy or binding profile across nonhuman homologues remains poorly documented in the literature. Our study also provides insights into the conformational changes induced by inhibitor binding over time, which are crucial for understanding the binding mechanism of VER-155008 against Hsp70, which could be useful to create more selective inhibitors that effectively target Hsp70s cancer-related functions while minimizing effects on Hsc70.

## Supplementary Material



## Notes

Supporting Information S1—the graph shows the conformational fluctuation profile per residue (RMSF, in Å) of an Hsp70 protein in the three in silico simulation conditions: bound to the inhibitor VER-155008 (blue line), bound to ADP (red line), and in the apo form (green line). Higher RMSF values indicate more flexible/mobile regions; low values indicate more rigid/stable regions. Supporting Information S2—RMSF per Hsc70 residue in the three conditions studied. Curves represent the average RMSF (Å) along the path for Hsc70 bound to the inhibitor VER-155008 (blue), bound to ADP (red), and in the apo form (green). Axes: residue number (*x*) and RMSF in Å (*y*) (PDF)
