# Supplementary material for: Unraveling the Molecular Inhibition and Conformational Changes of Hsp70 and Hsc70 Induced by VER-155008, a Competitive ATPase Inhibitor through Molecular Dynamics Simulations and Principal Component Analysis
Source: ACS Omega. 2025 Dec 17;10(51):62529–42. doi: 10.1021/acsomega.5c05832 (PMC12756766; doi:10.1021/acsomega.5c05832)
Supplement: Supplementary file 1 [file ao5c05832_si_001.pdf]

Supporting Information

Unraveling the Molecular Inhibition and Conformational Changes of Hsp70 and Hsc70 Induced by ver-155008, a Competitive ATPase Inhibitor through Molecular Dynamics Simulations and Principal Component Analysis

Maria Caroline Barbosa da Silva[1], Carlos Gefferson Silva Falabelo[2], Elvis Santos Leonardo[3], Claudir Oliveira[1], Renan Patrick da Penha Valente[4], Anderson H. Lima[4], Sérgio A. de Souza Farias[2], Khayth Nagata[2], Kauê Santana da Costa[1,*], and Paulo Sérgio Taube[1,*]

1 Universidade Federal do Oeste do Pará, Laboratório de Simulação Computacional, Instituto de Biodiversidade, Rua Vera Paz, Salé s/n, 68040-255 Santarém - PA Brazil.

2 Universidade Federal do Oeste do Pará, Laboratório de Simulação Computacional, Instituto de Ciências da Educação, 68040-255, Santarém, Pará, Brazil.

3 Laboratory of Computational Simulation and Molecular Modeling, Instituto Nacional de Pesquisas da Amazônia (INPA), 69067-375, Manaus, Amazonas, Brazil

4 Universidade Federal do Pará, Laboratório de Planejamento e Desenvolvimento de Fármacos, Instituto de Ciências Exatas e Naturais, Rua Augusto Corrêa, 01 Belém, 119, BR 66075-110

[*]Corresponding authors

Kauê Santana da Costa. E-mail: kaue.costa@ufopa.edu.br

Paulo Sérgio Taube. E-mail: pstjunior@yahoo.com.br

Supporting information 01 - The residue-wise conformational fluctuation profile (RMSF) of Hsp70 reveals clear differences in dynamics between the three states studied: bound to the inhibitor VER155008, bound to ADP, and in the apo form. In general, the APO condition has the lowest RMSF values, indicating a globally more rigid state, while the ADP condition shows increased mobility in several regions, notably along residues ~240–310, suggesting that ADP binding favors conformational movements associated with the functional cycle of the protein. The presence of VER155008 causes a marked peak in flexibility around residue ~100, greater than in the other states, which may reflect an allosteric effect of the inhibitor on local dynamics and possibly on communication between domains. Regions with consistently low RMSF (~0.8–1.2 Å) correspond to structurally stable segments, while pronounced peaks indicate mobile loops or handles that may participate in interactions or conformational transitions. These findings are consistent with a model in which distinct ligands modulate the local and global plasticity of Hsp70; it is recommended to map the peaks in the three-dimensional structure and complement with analyses (PCA, DCCM, replicates) to confirm the significance and functional repercussions of the observed differences.

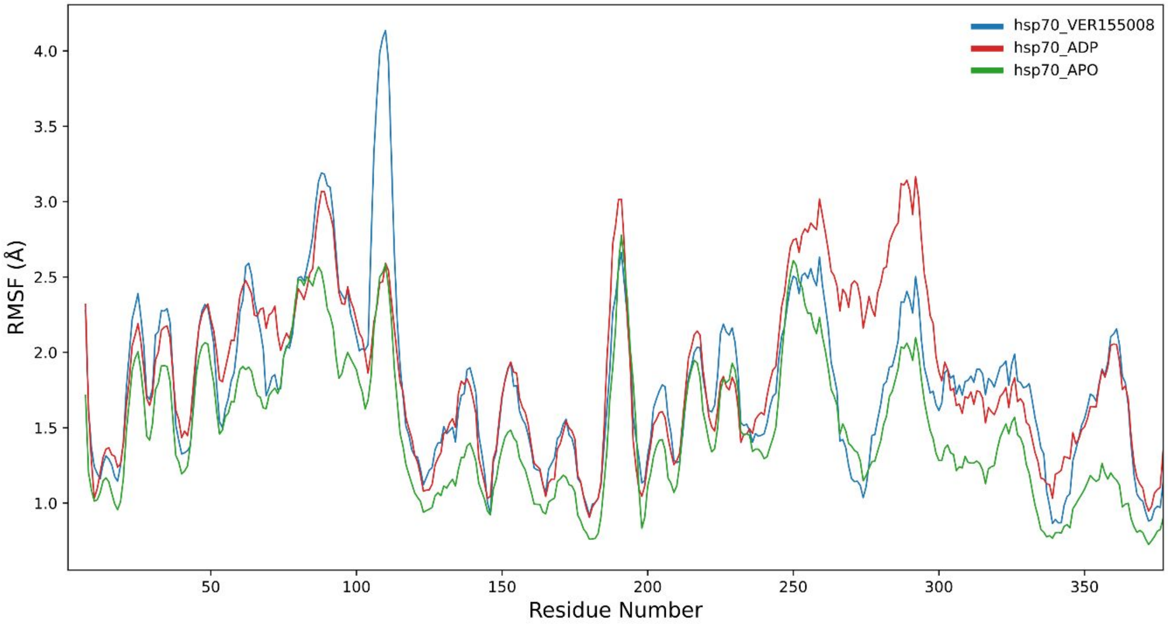

Figure S1 - The graph shows the conformational fluctuation profile per residue (RMSF, in Å) of an Hsp70 protein in the three in silico simulation conditions: bound to the inhibitor VER155008 (blue line), bound to ADP (red line), and in the apo form (green line). Higher RMSF values indicate more flexible/mobile regions; low values indicate more rigid/stable regions.

Supporting information 02 - The residue-wise conformational fluctuation profile (RMSF) of Hsc70 shows clear differences in dynamics between the VER155008, ADP, and APO states. In general, the apo form has the lowest RMSF values across most of the sequence, indicating a relatively more rigid state. The ADP condition exhibits increased mobility in several regions, particularly in the range of residues 240–310, suggesting that ADP binding favors conformational movements that may be relevant to the functional cycle of the chaperone. The presence of the VER155008 inhibitor is associated with a pronounced flexibility peak near residue ~100 (higher than in other conditions),

which may reflect a local allosteric effect of the inhibitor on the dynamics and communication between domains.

Regions with consistently low RMSF (approx. 0.8–1.2 Å) correspond to stable structural elements (probably helices/sheets), while marked peaks indicate mobile loops or handles that may participate in interactions with ligands/co-chaperones or conformational transitions.

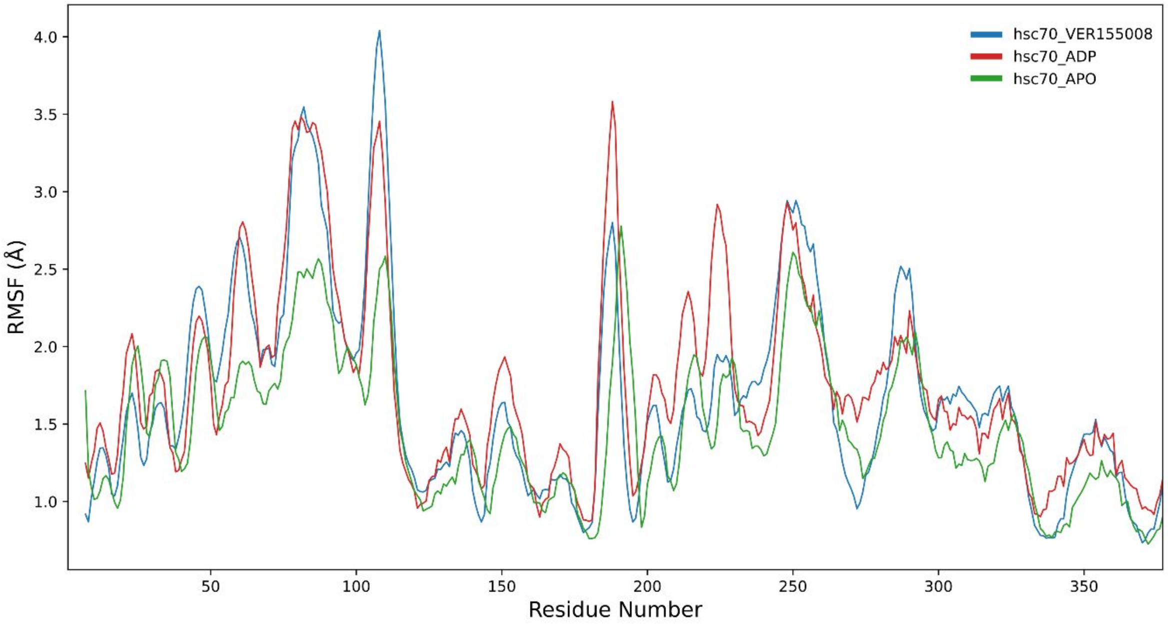

Figure S2 - RMSF per Hsc70 residue in the three conditions studied. Curves represent the average RMSF (Å) along the path for Hsc70 bound to the inhibitor VER155008 (blue), bound to ADP (red), and in the apo form (green). Axes: residue number (x) and RMSF in Å (y).
